# Supplementary material for: Are carbon emissions trading and green financial instruments synergistic? -Comprehensive quantitative research based on content analysis
Source: PLoS One. 2024 Mar 7;19(3):e0298601. doi: 10.1371/journal.pone.0298601 (PMC10919601; doi:10.1371/journal.pone.0298601)
Supplement: S1 Data — (ZIP) [file pone.0298601.s001.zip › Data/地级市Z_F工作报告.zip!/░▓╗╒/╦▐╓▌╩╨/└┤╫╘╣1⁄2╓┌║┼í╛┬φ┐╦╩2╛▌═°í┐_2014.docx]

2014年1月16日在宿州市第四届人民代表大会第三次会议上

市长　　史 翔

各位代表：

现在，我代表市人民政府，向大会报告政府工作，请予审议，并请市政协委员和其他列席人员提出意见。

一、2013年工作回顾

刚刚过去的一年，在市委的坚强领导下，全市上下深入贯彻党的十八大和十八届三中全会精神，攻坚克难，主动作为，经济社会发展稳中有进、稳中有为、稳中提质，较好完成了市四届人大一次会议确定的主要目标任务，实现了本届政府的良好开局。

初步核算，全市生产总值首超千亿元，增长11%左右；财政收入首超百亿元，增长20.8%，增速居全省第二；城镇居民人均可支配收入21705元，增长10%；农民人均纯收入7560元，增长14%；三次产业结构调整为24.8:41.7:33.5。“千百亿”目标顺利实现，动态指标保持全省先进，发展总量和速度同步提升，开启了我们加速崛起的新征程。

一是实体经济进一步壮大。规模以上工业增加值增长15%左右，规模以上工业企业近千家，工业产值超千亿元。加强企业帮扶指导，清理涉企收费项目181项，为企业减负30%以上。持之以恒打造轻纺食品、煤电能源、现代通信及应用三个千亿元产业基地。园区发展成为重要增长极，市经开区发展提速，产值过亿元企业达40家；中国现代制鞋产业城入驻项目近百个，规模以上工业产值43亿元；宿马园区一马当先，入驻项目45个，绩效考核名列皖北共建园区第一；高新区获准筹建，华为、世纪互联等项目投入运行。高新技术产业产值突破110亿元。节能减排完成省控目标。44个乡镇工业增加值超亿元，36个乡镇财政收入超千万元。工业发展已经跻身全省中等行列。

二是投资消费进一步扩大。超额完成省“861”建设任务，固定资产投资760亿元，增长25%以上，其中工业投资超430亿元，增长20%以上。259个亿元以上项目开工建设，鸿星尔克、冠军建材、惠丰新能源、海螺二期、协和风电、金黄庄煤矿、吉美纸业、鸿丰生物等96个亿元以上项目建成投产。积极承接转移，狠抓招商引资，实际到位内资1100亿元，利用外资4.7亿美元，进出口总额突破5亿美元。深化与央企、省企、知名民企合作，引资220亿元以上。全年贷款增量过百亿，创历史新高，灵璧农商行开业，交通银行宿州分行、市农商行获批筹建。加快现代服务业发展，南翔恒泰商贸物流城开工建设，大润发、希尔顿逸林酒店开业，社会消费品零售总额300亿元，增长14%。有效投入的快速增长，蓄积了全市跨越赶超的强大能量。

三是现代农业进一步提升。实现粮食生产“十连增”，畜禽生产“七连增”。现代农业产业联合体发展到68家、农业企业3174家、农民专业合作社5132家、家庭农场1317家、职业农民3639人，走在了全省前列。农产品加工产值突破800亿元，市级以上龙头企业发展到326家，砀山水果加工区跻身国家产业化示范基地。淮水北调工程开工建设，解决34.2万人饮水安全问题，埇桥、泗县、灵璧成为全国高标准农田水利建设示范县，埇桥荣获“江淮杯”一等奖。萧县成为全国改革创新试点县。实施千万亩森林增长工程，新造林16.3万亩。在全省率先启动人地挂钩试点，全国首个土地流转信托项目落户埇桥区帝元公司。农业大市向农业强市转变的步伐进一步加快。

四是城乡建设进一步提速。全市221项重点工程完成投资199亿元。泗许高速二期主体完工，宿淮铁路建成通车，宿灵泗等3条快速通道建设稳步推进。大外环建设持续加快，洪河路新汴河大桥即将通车，市规划馆主体框架完工，文化艺术中心基本建成。四个县城新区建设亮点纷呈，“五个一”工程进展良好，四项任务基本完成。美好乡村建设步伐加快，费村等36个省级示范村面貌焕然一新。完善征迁控违政策机制，全市完成征迁拆违670万平方米，其中市区超240万平方米，为历年之最。市城投集团顺利组建，全年融资40亿元。创新城市管理机制，争创省级文明城市，根治非法营运三轮车，城区增绿900万平方米，建成绿道示范段20公里，市容市貌有了明显改观。65平方公里建成区功能日益完善，200平方公里城市框架逐步拉开。今日的宿州已经发展成为初具规模的中等城市！

五是民生民计进一步改善。全年民生支出178亿元，占财政支出的80%，33项民生工程建设任务全面完成，10件惠民实事顺利实施。兴建城市节点游园16处，自来水一户一表改造5076户，新覆盖集中供暖居民3.5万户，新建“四有”社区12个，新汴河景观工程稳步推进，清水工程改造疏浚17公里。新建各类保障房3.3万套，基本建成1.7万套，综合考评居全省第一方阵。城镇登记失业率控制在3.9%以内，“五险”参保人数175万人，城乡居民社会养老保险全覆盖，城乡低保、五保供养标准提高10%以上，新农合参合率达98.3%，救助孤儿3689名、贫困患病群众6.5万人次，残疾人康复中心建成使用，城镇医保实现异地就医联网即时结算。人民群众实实在在感受到，生活年年都有新改善。

六是社会事业进一步发展。新建、改扩建公办幼儿园100所，完成257所城乡义务教育标准化学校建设，组建宿城第一初级中学。基层医药卫生体制改革成果巩固，12家县级医院实现药品零差率销售，市立医院通过三甲评审。WTA宿州国际女子网球公开赛成功举办，我市运动员王春雨勇夺亚锦赛田径金牌，全运会获得2金2银1铜。大型原创动画片《楚汉风云》在央视首播。话剧《石乡党魂——柯增华》晋京赴省演出圆满成功。灵璧磬云山地质公园确定为国家地质公园。人口计生工作取得突破，成功承办全国计生协组织建设工作会。食品药品安全监管规范有序。安全生产形势总体平稳。深入推进“书记带头大走访”，信访秩序持续好转。严厉打击各类违法犯罪活动，命案积案侦破全省第一。国防动员、人民防空和双拥工作成绩显著。各县区首次全部通过国家科技进步目标考核。第三次全国经济普查、“六五”普法顺利开展。民族宗教、外事侨务、防震减灾、旅游、物价、质量、扶贫、对台、烟草、气象、档案、工会、共青团、妇女儿童、老龄等各项工作取得全面进步。

在推进经济社会发展的过程中，我们始终高度重视政府自身建设。认真学习贯彻习近平总书记系列讲话精神，严格执行中央八项规定，开展党的群众路线教育实践活动前期准备工作。修订完善市政府工作规则，政府运行机制日趋规范。坚持经济社会发展一月一调度，强化工作落实。深入开展“点燃激情、提升效能”活动，实施“泗县赶泗洪、宿州学宿迁，泗县突破、全市跟进”，掀起效能建设新高潮。加大政府工作创新，推进简政放权，深化行政审批制度改革，市级行政审批事项减少37.6%，对高新区、宿马园区分别下放权限166项、172项。自觉接受人大法律监督、政协民主监督和社会监督，办理人大代表议案5件、建议119件、政协委员提案311件。强化行政监察和审计监督，勤俭办事，严禁新建楼堂馆所，以政风建设的新成效取信于民。

一年来的奋斗历程，带给我们许多深刻启示，也为今后发展积累了宝贵经验：我们必须牢固坚持整体承接、双轮驱动、三区共进、四化同步，坚定发展自信、路径自信、模式自信，汇聚齐心协力谋发展的磅礴力量；必须牢固坚持“抓主抓重、苦干实干、提速提效、争先争优”16字要求，坚持思想同心、目标同向、行动同步，形成一心一意干事业的生动局面；必须牢固坚持敢闯敢试、敢于担当的政治勇气，着力推进改革，向改革要动力、要效率、要红利；必须牢固坚持以人为本、民生优先的执政理念，时刻关注民情、服务民生，不断提高群众生活质量和幸福指数；必须牢固坚持“点燃激情、提升效能”的工作要求，弘扬“五加二”、“白加黑”的作风，提振全市上下干事创业的精气神，在抢抓机遇中推动发展，在应对挑战中争先进位。

成绩来之不易，经验弥足珍贵。这是市委总揽全局、坚强领导的结果，是市人大、市政协和社会各界监督支持的结果，更是全市上下同心协力、顽强拼搏的结果。在此，我代表市人民政府，向奋战在各行各业的全体劳动者，向离退休老同志，向各民主党派、工商联和人民团体，向中央和省驻宿单位、人民解放军驻宿部队、武警官兵、公安干警，向参与宿州建设的海内外客商、广大援宿干部，以及所有支持宿州发展的各界人士，表示衷心的感谢，并致以崇高的敬意！

回顾过去的一年，我们要清醒认识到，经济社会发展中还存在不少困难和问题。我市在全省经济总量居中、人均水平靠后的格局没有根本改变，受经济下行态势影响，个别指标没有达到预期目标；工业化水平不高，城镇承载能力不强，主导产业特色不鲜明，县域经济发展严重滞后；要素制约日趋加紧，战略性新兴产业规模偏小；公共服务和社会保障水平不高，少数群众生活困难；政府职能转变还不到位，“四风”问题和腐败现象仍然时有发生，机关效能建设尚需持续推进，发展环境有待进一步优化。对此，我们一定高度重视，采取措施，有序化解，不辜负全市人民的希望和重托。

二、2014年的主要工作

2014年是贯彻落实十八届三中全会精神、全面深化改革的重要一年，也是实现“十二五”规划目标任务的关键一年。做好今年政府工作，意义重大，影响深远。综合判断，我市仍处于可以大有作为的黄金发展期，工业化中期快速推进的阶段性特征更加鲜明，双轮驱动积蓄的能量更加强劲，中原经济区、中部崛起、皖北振兴政策叠加效应更加凸显，深化改革推动发展的空间更加广阔。同时要看到，我国经济进入增长速度换挡期、结构调整阵痛期交织阶段，发展面临的外部环境更加复杂多变，前进道路上还有更多的挑战和困难。对此，我们既要做好应对困难局面的准备，又要坚定发展信心，牢固树立战略思维和底线思维，紧扣宿州实际，扎实开展工作，全力推动经济社会又好又快发展。

今年政府工作的总体思路是：全面贯彻党的十八大、十八届三中全会和中央、全省经济工作会议精神，毫不动摇坚持以经济建设为中心，坚持稳中求进、改革创新，坚持整体承接、双轮驱动、三区共进、四化同步，实施“全市工业和城乡建设三年振兴计划”，持续扩大有效投入，大力发展实体经济，唱响县域经济主旋律，在建设“实力宿州、活力宿州、和谐宿州、幸福宿州”征程上，迈出新的更加坚实的步伐。

经济社会发展主要预期目标是：全市生产总值增长11%左右，财政收入增长15%左右，固定资产投资增长20%以上，规模以上工业增加值增长15%左右，社会消费品零售总额增长13%，城镇居民人均可支配收入增长11%以上，农民人均纯收入增长14%以上，城镇登记失业率控制在4.5%以内，节能减排完成省控目标，主要动态指标继续位居全省前列。重点做好八个方面工作：

（一）强化指导扶持，加快振兴县域经济。把县域经济发展摆上更加突出的战略位置，以“全市工业和城乡建设三年振兴计划”为核心，在全市范围内唱响县域经济的主旋律。

彰显县域产业特色。振兴县域经济，是我市今后一个时期的核心任务，而县域经济的“发动机”，就是工业主导产业和产业集聚。各县区要精准定位、错位发展，加快首位产业培育，构筑特色产业体系，全省争位次，皖北争上游。实施“3111”行动计划，各县区、各园区通过3年努力，打造1个在全国叫得响的首位工业产业，实现产值超100亿元，各县区、各园区实现工业税收超10亿元，其中首位工业产业税收超5亿元。支持埇桥区做大纺织服装产业，砀山县打造果蔬食品产业，萧县壮大食品医药产业，灵璧县发展轻纺食品产业，泗县做强机械电子产业。引导有条件的乡镇发展镇域经济、村集体经济，努力形成一镇一业、一村一品。

制定完善配套政策。坚持事权财权一致，进一步下放市级经济社会管理权限，凡是能够下放给县区的，全部清理、下放到位。建立支持县域经济发展的长效机制，加强对县区重大基础设施、公共事业建设的财政支持。着力解决县域发展要素制约，出台金融业支持县域经济发展、首位产业培育的实施意见，鼓励商业银行扩大信贷投放规模、下放支行业务权限，完善担保再担保体系，不断加大担保机构支持县域经济发展的力度；积极争取省预留用地和点供地指标，用足用好城乡建设用地增减挂钩政策，把用地指标与县域重大工业项目相挂钩。

加大考核奖惩力度。完善县域经济考核体系，加大对县区工业、园区和城乡建设相关指标的考核权重，形成奖优罚劣、奖勤罚懒的良好导向。继续坚持县域经济一月一调度，强化对县区首位产业的专项调度，全面激发县域经济自主发展活力。

（二）聚焦工业引领，加快推进工业化进程。把支持实体经济作为关键举措，把扩大有效投入作为重要抓手，确保规模以上工业增加值实现320亿元。

打造主导产业。坚定不移推进工业总量扩张、结构优化，形成主导产业顶天立地、全民创业铺天盖地、新兴产业后来居上之势。以打造轻纺食品、煤电能源、现代通信及应用三大千亿元产业基地为目标，着力发展纺织鞋服首位产业，加快鑫国龙鞋业、野力体育、七匹狼和宝德二期、国口酒业、海升果胶等项目建设；着力推进国电龙源风电、祁县低热值煤发电、深能集团符离光伏发电等项目，力争钱营孜低热值煤发电项目开工；着力加快智慧云计算产业园、中科云智慧产业园建设。推动战略性新兴产业集聚发展，确保欣意电缆、华林电子、佳力奇碳纤维等项目建成投产。

主攻工业招商。以“工业项目、招大引强、税源企业和环境保护”为原则，创新招商引资工作，全市实际到位内资增长20%，实际利用外资增长15%以上。继续实施全民招商，实施无会周外出招商、领导带头招商、招商任务计划公开和离岗招商等制度，同时将深入开展产业招商、专业招商、链式招商，成立园区专业招商局、招商专业小分队。围绕首位产业和主导产业培育，瞄准产业链上、中、下游和左右配套产业，紧盯旗舰型企业，坚持龙头引进、整体承接，形成产业集聚。继续深化与央企、省企、知名民企新一轮合作。坚持市县区联动招商，强化信息共享，建立全市招商信息和项目落地共享平台。坚持以税收和就业实绩检验招商实效，完善考核、奖惩办法，加大招商有功人员奖励力度。严格执行项目投资强度、投入产出率、税收贡献率等准入标准，坚决防止投机圈地和引进落后产能。

推进项目和园区建设。坚持经济工作项目化、项目工作责任化、责任追究制度化，推进重大项目提速提效，力争固定资产投资突破900亿元。实行腾退机制，提升“5161”项目库质量，储备规模万亿元以上。争取列入省“861”项目330个，年度投资超220亿元。重点组织实施亿元以上项目，力争年度投资500亿元，其中10亿元以上项目50个。完善市级、县处级领导干部联系项目制度，按照“五个一”的要求，强化重大项目密集调度、定期通报，提高重大项目的开工率、竣工率、达产率。加快各类园区建设，市经开区突出发展生物医药首位产业，加快推进亿帆药业、皖淮生物、安特生化等项目，实现规模以上工业增加值39亿元，积极创建国家级开发区；制鞋产业城一期建成，功能基本完善，实现产值超60亿元、税收超6亿元，加快向“3311”目标迈进；宿马园区加快推进苏湘高新产业园等项目建设，争当全省一流的共建园区；高新区重点发展云计算、家居和动漫三大主导产业，着力打造高端高效园区；各县区开发区力争扩区3平方公里，鼓励开展形式多样的园区合作共建。

（三）重视功能完善，加快推进城乡建设。坚持双轮驱动，推动以人为核心的新型城镇化，全年实现投资220亿元，实施210个重点项目，全市城镇化率提高2个百分点以上。

突出中心城市带动。围绕城市总体规划，进一步完善单元规划，加快控制性详规、片区规划编制，优化专项规划体系，对园区规划实施总体控制、局部放权。坚持东向高铁、北跨汴河、南展新城、西越高速，强力推进主城区、汴北新区、城南新区、城东新区、高铁新区五大片区建设，实现“双70”目标。加快实施路网工程，启动西环线等过境循环路网建设，加快大外环、拱辰路等建设进度，建成南北环公跨铁立交桥。加快实施保障性安居工程，新建各类保障房2.46万套，实施棚户区改造1.8万套。推进依法和谐征迁，交付净地1000亩。授权埇桥区对道东区域进行建设经营管理。

加强县城和小城镇建设。开展县城规划建设管理水平提质达标行动。以完善基础、配套功能、提升品位、打造特色为核心，全面实施“555”工程。在县城建设层面，四个县各建设一个不低于10万平方米的商业综合体，一处不低于10万平方米的保障性安居工程，一处全民健身运动中心，一个不低于5000平方米的标准化集贸市场，改造提升一条不少于500米的绿化亮化街区。在镇村发展上，各县区均新增2个以上工业增加值过亿元、财政收入过千万元的“双超”乡镇，新增10个以上集体经济收入过10万元的村，新增7个美好乡村示范村，每个乡镇完成3万平方米的征迁控违任务，五个县区共新增土地流转面积70万亩以上。在工作要求上，各县区均完成土地出让收入10亿元，收储土地1000亩，完成一个镇当县建的总体规划，征迁整理净地不低于500亩，每个县城扩区3平方公里。

完善城乡功能。加快推进济祁高速、郑徐客运专线建设，确保徐明高速建成通车，积极做好宿州机场等前期工作，力促符夹复线、京沪高铁宿州东站扩建工程开工建设。投资18.3亿元，全面开工宿灵泗、宿萧砀、206国道快速通道等一级公路项目，增强城乡联系的紧密度。新建市汽车客运中心站、京沪高铁客运枢纽站，改造升级县乡公路120公里，改造加固农村公路危桥140座。加快供水、供气、供暖等各类管网建设，重点推进汴北水厂、污水处理厂、垃圾处理厂等项目，完善加气站及配套设施，实施城乡电网建设改造。加快4G网络建设，推进智慧城市发展。

提升城镇品位。扎实开展绿化大行动，增量提质，破硬增绿，增加沿路临水绿化工程，城区新增绿化面积超500万平方米。建设城市绿道40公里。继续推进千万亩森林增长工程，加快建设交通干线两侧林苗两用林，完成人工造林14.3万亩。继续实施城乡增绿工程，加快建设园林景观，重点推进城市森林公园、野生动物园、三角洲公园改造提升工程建设，争创国家级园林城市。积极引进全国知名企业，建设高端商业综合体，提升商品房品质。巩固省级文明城市创建成果，注重向农村、向城乡结合部、向千家万户、向旅游景点延伸，建立长效机制。继续整治车辆乱停乱放，规范管理户外广告、施工围墙、渣土运输等，全力提升城乡“序化、净化、绿化、亮化、美化”水平。成立城市管理委员会，建立大城管工作格局。

（四）突出三农工作，加快发展现代农业。坚持以发展现代农业为主线，以美好乡村建设为重点，以增加农民收入为目标，大力促进农业农村经济平稳较快发展。

促进现代农业发展。统筹推进“两区”建设，抓好20个示范点，规划建设10万亩现代农业示范核心区。加快发展新型农业经营主体，现代农业产业联合体发展到100家，新增农业企业100家、农民专业合作社300家、家庭农场500家、职业农民2000人。重点推进泗县现代农业物流中心、砀山和萧县强英鸭业产业园、灵璧现代农业博览园、埇桥西二铺都市农业示范园等项目建设，力争现代农业重点项目投资规模突破85亿元，新引进亿元项目10个以上。推进农业清洁生产，加快市级农产品检测中心建设，强化农产品质量安全。创新农村土地经营制度，在农民自愿前提下，引导土地向新型农业经营主体流转。加快农业产业化进程，实现农产品加工产值900亿元，市级以上龙头企业达400家，超亿元企业150家。加快推进粮食生产全程机械化，建设45个万亩高产创建示范片，力争粮食总产400万吨以上。

加快美好乡村建设。全面修编完善县域村庄布点规划、村镇建设改造规划，新建37个省级重点示范中心村。加强农村基础设施建设，完善建管新机制。全面完成农田水利三年提升工程建设任务，新增灌溉面积83万亩。积极推进新汴河治理、淮水北调等重点水利工程建设，完成7条中小河流治理，除险加固19座小水库，解决45万人饮水安全问题，推进采煤沉陷区大型水库、怀洪新河洼地治理项目前期工作。

推进“三线三边”环境治理。市财政安排5000万元专项资金，以铁路沿线、公路沿线、河流沿线及城市周边、省际周边、景区周边为突破口，大力实施“四治理一提升”，推进农村环境综合整治。集中整治脏乱差，建立“户集中、村收集、镇转运、县处理”的农村生活垃圾处理模式，逐步完善垃圾污水处理长效机制。

提升农民生活水平。落实各项强农惠农政策，着力构建多元化农民增收支撑体系。加快实施农民就业创业工程，开展“一村一名实用技能人才”免费培训，创建省级农民工创业园2家。深化农村综合改革，加快推进农村土地确权登记，加大对产业联合体、家庭农场的金融产品创新，探索开展农村土地承包经营权、宅基地使用权抵押贷款试点，抓好土地信托新模式试点，推进粮食和特色农产品保险提标扩面。全力整合资源，加强扶贫政策的衔接和落实，大力开展产业扶贫，认真实施扶贫项目，打好扶贫攻坚战。

（五）扩大消费需求，加快提升现代服务业。坚持生产性服务业、生活性服务业协同并进，推动服务业发展提速、比重提高、结构提升，服务业增加值增长10%。

大力发展商贸物流业。加强大型商贸中心、配送中心和综合性商品交易市场建设，加快国购广场、红星美凯龙、南翔恒泰、华洋新世界国际广场建设。依托交通区位优势，做大做强骨干流通企业，推动营销网、物流网、信息网有机融合，开工建设林安物流，新增限上商贸流通企业40家。积极发展新型消费业态，促进市场持续繁荣。加快社区服务设施建设，打造15分钟便民服务圈。深入推进“万村千乡市场工程”，建设农村物流配送站，鼓励发展乡镇商贸中心，引导城市商业网点向农村延伸，构建城乡商品流通双向渠道。

积极发展新兴服务业态。加快发展科技、电子商务、总部经济、金融、信息等服务业，鼓励中小流通企业开展网上交易。围绕主导产业的服务配套，加大生产性服务业招商引资力度，开工建设新濠畔会展中心、鸿星尔克电商中心、苏宁云商等项目。抓好埇桥区服务业综合改革试点，积极申报省级服务业集聚区。

（六）深化改革开放，加快释放发展活力。今年是改革之年，必须认真贯彻中央全面深化改革的决策部署，突出抓好经济体制改革这一重点。

扎实推进各类改革。深化国有企业改革，完善国有资产管理体制，积极发展混合所有制经济。促进财税体制改革，改进预算管理制度，年内力争实现县级以上部门预算全部公开。继续做好“营改增”扩围试点。采取贴息、担保、后奖补等方式，安排财政支持企业发展资金。逐步将政府性债务收支分类纳入预算管理，严格举债程序。加快金融改革创新，大力培育地方法人金融机构，加快引进股份制商业银行，全面完成农村合作金融机构改革任务，实现徽商银行、村镇银行县域全覆盖。加快发展直接融资，推动上市后备企业尽快在主板及场外市场挂牌上市。以市城投集团为龙头，整合交投、工投、教投等各类融资平台，实施统贷统还、分类使用、财政监管、带动各县，壮大政府融资平台。加快建设信用体系，全面落实与各金融机构战略合作协议，全年贷款增量力争达到115亿元。做大市土地储备发展中心，提高土地保障能力，发挥融资职能。强化节约集约用地，积极稳妥推进人地挂钩试点，加快建立城乡统一的用地市场。

大力发展民营经济。不断优化发展环境，深入贯彻省“20条”、市“27条”发展民营经济意见，安排6000万元专项扶持资金，支持民营企业健康发展，激发全民创业潮，新增私营企业2200户。坚持问题导向，探索建立负面清单管理模式，全面清理、废除各种不合理规定和隐形壁垒，坚决打破“玻璃门、弹簧门、旋转门”，推进工商注册制度便利化，由先证后照改为先照后证，提升民营企业综合服务水平。加快推进海关、国检设立正式机构，建设中科云智慧保税仓库，抓好进出口企业孵化工作，进出口总额增长15%。总之，要让民营经济在我市遍地开花、茁壮成长。

（七）创新社会治理，加快构建和谐宿州。改进社会治理方式，推进社会事业改革创新，切实维护人民群众权益，保持社会安定有序，确保人民安居乐业。

大力推进文化建设。高举中国梦的伟大旗帜，深入做好文明创建工作，开展宿州好人评选、志愿服务等活动。实施文化惠民工程，加快市县文化场馆等设施建设，广泛开展群众性文化活动，继续推进农村电影放映提质、百戏进百乡。深化文化体制改革，实施文艺精品工程，塑造宿州文化知名品牌。继续推动大运河申遗保护。大力发展文化旅游产业，加快神游世界动漫产业园、黄河故道综合开发、五柳景区、大方寺、钟馗文化园等项目建设，推进皇藏峪5A级景区创建。全年接待国内外游客1100万人次，旅游总收入超75亿元。

统筹发展社会事业。实施全市义务教育校舍维修改造，完成167所标准化学校建设，学前三年入园率达80%以上。强化校园安全管理，综合整治校园周边环境。加强教师队伍建设。支持宿州学院、职业技术学院发展，加快教育园区建设，完成逸夫师范学校新校区一期建设，加快市职教中心一期建设。巩固完善县级公立医院综合改革，加快市立医院和萧县、泗县、灵璧县医院新院区建设，提升医疗服务质量。落实重大疾病防控措施，加强卫生应急能力建设，积极发展中医药事业。广泛开展全民健身运动，积极备战省第十三届运动会，建成开放市体育馆。实施创新驱动战略，启动科技“小巨人”计划，加速科技成果转化。着力加强污染综合治理和生态保护，严格执行节能减排一票否决制，启动市、县级细颗粒物检测能力建设，加强秸秆综合利用和禁烧工作，强化饮用水水源地保护。严格落实人口计生目标责任制，启动实施“单独两孩”政策。高度重视第三次全国经济普查，继续实施“六五”普法，加强国防动员、人防和民兵预备役建设，扎实做好民族宗教、档案、残疾人、红十字、防震减灾、外事侨务和对台工作，协调推进供销、气象、烟草等工作。

持续维护社会和谐。加强食品药品安全监管，实施安全溯源管理，严厉打击不合格食品制售等违法行为。认真落实安全生产责任制，强化煤矿、非煤矿山、道路交通、建筑施工、消防、危险化学品监管，建立隐患排查治理和安全预防控制体系，严防重特大事故发生。严格执行车辆超限超载治理条例，加大依法“打非治违”力度。新增“四有”标准社区10个，推进农村社区建设试点工作。创新群众工作方法，全面推行联合接访，落实领导包案责任制，强化基层矛盾排查化解，畅通、规范群众诉求表达渠道。切实加强应急管理，努力提升突发事件防范、应对和处置水平。推进立体化、数字化社会治安防控体系建设，加大依法管理网络力度，严密防范和惩治各类违法犯罪活动，加快建设平安宿州、法治宿州。

（八）改善民生民计，加快建设幸福宿州。按照保基本、补短板、兜底线、可持续的要求，紧紧围绕群众最关心、最紧迫的民生需要，多谋民生福祉之事，让全市人民共享美好生活。

完善社会保障体系。进一步加大财政保障力度，落实城乡养老保险制度统筹衔接办法，“五险”参保人数达到179万人次。努力提高优抚待遇，城乡低保标准、五保供养标准提高10%。调整实施城镇居民基本医疗保险政策，巩固基层医保覆盖面，新农合参合率达到95%以上。完善农村留守妇女、儿童、老人关爱帮扶体系。抓好价格监管，稳定民生价格。稳步推进城乡居民收入倍增规划，加大对低收入群体、特殊困难群体的社会救助力度。逐步把进城落户农民完全纳入城镇住房和社会保障体系，有序推进农业转移人口市民化。

多渠道推动创业就业。把保就业作为稳增长的底线，建立发展就业联动机制，一手抓援企稳岗，一手抓以创业带就业。实施高校毕业生就业促进计划，加强农村转移劳动力、城镇困难人员、退役军人等重点群体就业工作。鼓励引导劳动力就近就业创业，继续实施“农民工进城圆梦计划”，加快示范性创业孵化基地建设，积极申报国家级创业型城市。大力推进“十百千万”人才引进培养工程，分类建立人才库。完善工资集体协商制度，严格执行最低工资标准和农民工工资支付保障制度。

巩固提升民生工程。在实施33项民生工程基础上，本着“广覆盖、重实效、惠民生”的原则，再办10件惠民实事，让更多的群众享受改革发展成果。

1．全市新建、改扩建、增设附属公办幼儿园54所，规划建设道东初中

2．市区建成使用市120指挥中心，开工建设市妇幼保健所

3．开工建设市科技馆、游泳馆、妇女儿童活动中心、会展中心

4．更新市区公交车辆，优化公交线网

5．市区投放公共自行车1700辆，增建社会停车泊位3000个

6．全市规划建设各类便民专业市场32个

7．整治提升市区中山街商品市场

8．全市新建、提升改造一批标准水冲公厕

9．全市开工建设3～5座垃圾焚烧发电厂

10．继续实施新汴河景观工程、清水工程

三、进一步加强政府自身建设

要认真落实中央八项规定和省、市三十条意见，紧紧围绕为民务实清廉要求，全面正确履行政府职能，不断提升服务人民群众、加快科学发展的执政水平。

转变职能，建设创新政府。用改革的思路推进工作，不断创新举措、方法和路径，既要连续性，又要创造性。简政放权，凡是市场能办的一律交给市场，凡是社会能办的一律交给社会，把该放的权力放掉，把该管的事务管好。正确对待利益格局调整，深化行政审批制度改革，建立各部门行政审批事项清单制度，清单之外一律不设行政审批事项；进一步削减、下放、取消、规范审批事项，继续推进“四个一”工程，不断松绑、放权、减负、让利。优化政府机构设置、职能配置和工作流程，完成工商、质监管理体制调整和市县食品药品监管体制改革，组建县级市场监督管理机构，推进卫生与计划生育部门机构整合。加快事业单位分类改革，推进政府购买服务。

执政为民，建设务实政府。把促进社会公平正义、增进人民福祉作为一面镜子，审视政府工作，坚持求真务实，有面子、没里子的事不干，只顾眼前、不顾长远的事不干，与民争利、引起民怨的事不干。把群众路线作为根本工作路线，深入开展第二批党的群众路线教育实践活动，尊重人民主体地位，走进一线察民情，深入基层接地气，从群众中提炼破解难题的经验和方法，从基层中捕捉创新工作的思路和方向。牢记“功成不必在我，为政贵在有恒”，与人民心心相印、与人民同甘共苦、与人民团结奋斗。

永葆激情，建设高效政府。严格绩效管理，突出责任落实，奖优罚劣，追责问责，倒逼谋事干事的精气神。加强重大决策、重点工作、重要项目的推进，坚持周通报、月调度、季总结，环环紧扣抓落实。各级领导干部要率先点燃激情，深入一线，靠前指挥，雷厉风行干事业，身先士卒作表率。重拳整治“不作为、乱作为、慢作为”、“中梗阻、基层板结”，严厉查处推诿扯皮、效率低下等行为，严肃政令，改进作风，推动政府工作提速提效。

规范透明，建设法治政府。严格执行政府工作规则，依法行政，按规矩办事，扩大社会公众参与度，提高政府决策透明度和群众满意度。深化行政执法体制改革，严控自由裁量权，严格、规范、公正、文明执法。加强公共资源交易活动监督管理。自觉接受人大法律监督和政协民主监督，切实办好人大议案、代表建议和政协委员提案。深入推进政务公开和办事公开制度，加强政府信息公开平台建设，向群众说真话、交实底，让百姓看得懂、说得清。推行权力清单制度和行政复议制度，创造条件让人民监督政府，让权力在阳光下运行。

勤勉清廉，建设廉洁政府。全面落实党风廉政建设责任制，着力构建不敢腐的惩戒机制、不能腐的防范机制、不易腐的保障机制，增强全社会对反腐倡廉的信心。加强行政监察和审计监督，坚决整治损害群众利益的不正之风。集中解决“四风”问题，动真格、求实效。厉行节约反对浪费，严控公务活动成本。强化管理、严格自律，努力建设一支信念坚定、为民服务、勤政务实、清正廉洁的公务员队伍。

各位代表！

新的航程已经开启，前进的号角催人奋进。让我们紧密团结在以习近平同志为总书记的党中央周围，在市委的坚强领导下，进一步解放思想、锐意进取，同心同德、扎实工作，努力谱写宿州加速发展、奋力崛起的新篇章！
